# Discovery of *Ornithodoros tholozani*, the Main Vector of Iranian Tick-born Relapsing Fever

**DOI:** 10.34172/aim.31196

**Published:** 2024-10-01

**Authors:** Ali Emadzadeh, Shirin Taraz Jamshidi

**Affiliations:** ^1^Department of Internal Medicine, Faculty of Medicine, Mashhad Medical Sciences, Islamic Azad University, Mashhad, Iran; ^2^Department of Pathology, Faculty of Medicine, Mashhad University of Medical Sciences, Mashhad, Iran

**Keywords:** Joseph Tholozan, *Ornithodoros tholozani*, Relapsing fever

## Abstract

Doctor Joseph Désiré Tholozan was a French physician who became the special physician of Nassereddin Shah, King of Persia (Iran) in the 19^th^ century. He studied lots of topics in the field of epidemiology of infectious diseases. His efforts led to the discovery of the main vector of Iranian tick-born relapsing fever, *Ornithodoros tholozani*. He was also one of the pioneers of the Iranian Sanitary Council, whose efforts led to the foundation of the Ministry of Health of Iran a few decades later. This paper is a brief review of his biography and his roles in promoting health in Iran in the 19^th^ century.

## From an Island in the Indian Ocean to the Crimean War

 Born in Diego Garcia, Mauritius, in the Indian Ocean, in 1820, Joseph Désiré Tholozan was the son of a French couple.^1,2^ He entered primary school in Port-Louis, Mauritius.^2^ A few years later, the Tholozan family returned to Marseille, France.^2^ Joseph entered Marseille medical school. His uncle, Dr. Andre Francois Cauviere (1780-1858), was the director of this school at that time.^3^ He joined the French Military Health Service as an auxiliary assistant surgeon in 1841, while he was still a medical student.^3^ He obtained his medical degree (M.D.) in 1843 in Paris.^3^ His dissertation was about malignant metastases later dedicated to Nassereddin Shah Qajar, Shah of Persia, in the preceding years.^4^

 As a military physician, Tholozan then participated in the Crimean War (1854-55) in Napoleon III’s period.^3^ In that war, he faced troops suffering from infectious diseases such as typhus and cholera.^3^

**Figure 1:**
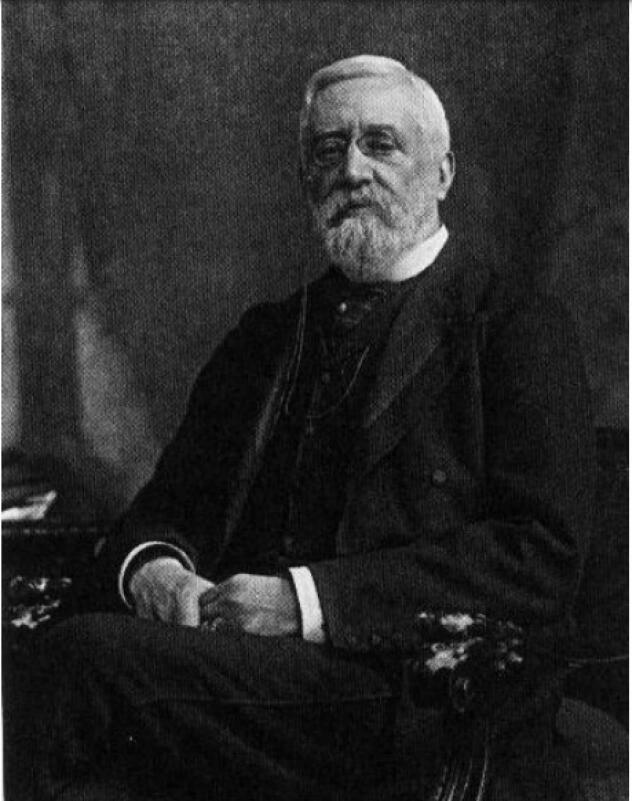
Joseph Désiré Tholozan (1820-1897), One of the Founders of the Iranian Sanitary Council and the Special Physician of Nassereddin Shah Qajar. *Source*. Adapted with permission from Azizi^5^

 In 1858, Farrokh Khan Ghaffari (Amin Al-Molk), the great ambassador of Persia (Iran) in the court of Napoleon III in Paris, requested the French Government to send an expert physician to Iran to become the personal physician to Nassereddin Shah Qajar, King of Persia.^4^ The Ministry of Foreign Affairs of France selected Doctor Joseph Désiré Tholozan for this purpose. Thus, Dr. Tholozan came to Tehran, the capital of Iran, and became the special physician of Nasseredin Shah instead of the Austrian physician, Dr. Jakob Eduard Polak.^4,5^ (Figure 1).

## From Paris to Tehran

 During Tholozan’s residency period in Iran, not only did he act as the special physician of the Qajar court, but he also treated patients from the general Iranian population. He studied diseases that were so common in Iran at that time. For instance, he gathered a dozen pieces of data about plague, cholera, and Iranian relapsing fever and scrutinized them,^3,4^ and recommended comments to promote general public health in Iran.^6^ Of those, his research on Iranian relapsing fever was highly important because it led to a great discovery in medical entomology that will be mentioned in the preceding sections of this paper.

## Dr. Tholozan and Relapsing Fever

 Relapsing fever is an arthropod-borne infectious disease, caused by spirochetes of the *Borrelia* genus. It is characterized by relapsing episodes of fever with simultaneous spirochetemia. Relapsing fever is subdivided into louse-born relapsing fever or epidemic relapsing fever and tick-born relapsing fever (TBRF) or endemic relapsing fever.^7,8^

 The *Borrelia* species that cause TBRF can vary depending on the geographic location; for instance, *Borrelia hermsii* and *Borrelia turicatae* are endemic in North America, but *Borrelia persica* is endemic in the Middle East (including Iran) and Central Asia.^9,10^ In the 19th century, TBRF was highly common in Iran, but its special vector was not obvious.

**Figure 2:**
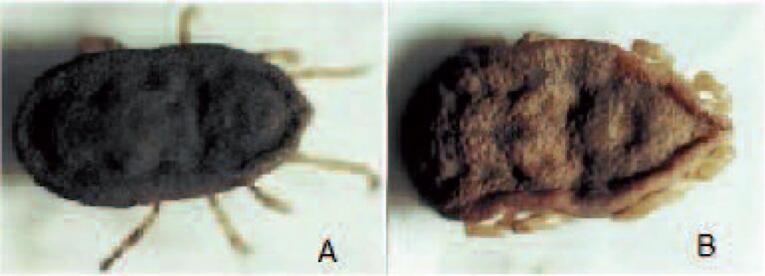
The Main Vectors of Relapsing Fever in Iran (A: *O. tholozani* and B: *O. lahorensis*). *Source*. Adapted with permission from Moradi-Asl and Jafari^12^

 Dr. Tholozan meticulously observed patients suffering from Iranian relapsing fever due to Borrelia persica. He collected ticks from his patients’ bodies and sent them to his colleagues in Europe.^11^ Among ticks he sent, Laboulbene and Megnin, two expert entomologists in France, found the true vector of Iranian TBRF and named it in honor of Dr. Joseph Tholozan as Argas (presently Ornithodoros) tholozani.^11,12^ (Figure 2).

## A Glance on Other Infectious Diseases Dr. Tholozan Studied in Iran

 Tholozan also studied the epidemiology of plague in Iran.^13^ This disease was endemic in Kurdistan, in the western part of Iran. Tholozan had already marked the foci of the disease in some well-defined villages and provided a detailed clinical description of the appearance of flea bites on the patients’ skin.^13^ In the book ‘Tauon’ (plague), written by Mohammad Razi Tabatabai, the chief of military physicians in Nassereddin Shah’s period, which must be the first Iranian medical book written about this disease in recent centuries, there are some writings of Tholozan about the plague.^14^

 It is so important to mention that Tholozan’s works on plague paved the way for Dr. Marcel Baltazard from the Pasteur Institute of Iran to study the epidemiology of plague in Iran about one hundred years later.^13^

## Dr. Tholozan’s Publications

 Tholozan published over fifty books and papers in his life period, mainly about infectious pathology and epidemiology,^1^ among which some of the academic books written by Tholozan are as follows^4^:

 “Zobdah Al-Hekmat of Nasseri” which is about the useful effects of *Cinchona *(in Persian).

 “Badaye Al-Hekmat of Nasseri” which delves into the auscultation of heart and lung sounds (in Persian).

 Prevention and Treatment of Plague (in Persian).

 Resaaley-e Tebb e Omoomi (in Persian): A Note on General Medicine.

 Des Métastases,Thèse présentée et soutenue á la faculté de médicine.Librairie de A. Delahaye et E. Chatel. Paris (Dissertation of Dr. Tholozan that was dedicated to Nasereddin Shah and is now lodged in the library of Baharestan^4^ (The Official Library of Iranian Parliament).

## Doctor Tholozan and Foundation of Sanitary Council in Iran

 One of the most impressive reputations of Tholozan in Iran is his collaboration in the foundation of a council that was called “Majles-e Hefz al-Sehheh,” which implies Health or Sanitary Council, which was the cornerstone of the foundation of the Ministry of Health of Iran in preceding decades.^4,5^ He became the first president of this council.^5^

 In his duty period in this council, he always looked upon measures imposed by international health societies regarding quarantine with suspicion. He believed that quarantining must be scheduled by native medical staff rather than foreign physicians who are unaware of the culture of the target societies.^6^

## Aftermath

 Tholozan practiced in Iran for 38 years, and due to his familiarity with the Persian language and Iranian culture, he had an impressive role in promoting health in Iran. Owing to his unique contribution to the court of the Qajar Dynasty, he was honored by the epithet “Hakim Baashi” by Nassereddin Shah; thus, it seems that he is the only non-Iranian physician who was honored by the Qajar court with this epithet.^4^ Finally, in 1888, Dr. Tholozan retired and returned to France, but soon later, he returned to Iran and stayed there until he died in 1897. He was buried at Doulab Cemetery, Tehran.^4,15^

 In conclusion, Dr. Joseph Tholozan’s great roles in the promotion of the epidemiology of infectious diseases in Iran, especially his great role in discovering the main vector of Iranian TBRF, and his impressive role in establishing “Majles-e Hefz al-Sehheh”, are unforgettable.
